# Supplementary figures and images for: AIM: A network model of attention in auditory cortex
Source: PLoS Comput Biol. 2021 Aug 27;17(8):e1009356. doi: 10.1371/journal.pcbi.1009356 (PMC8462696; doi:10.1371/journal.pcbi.1009356)

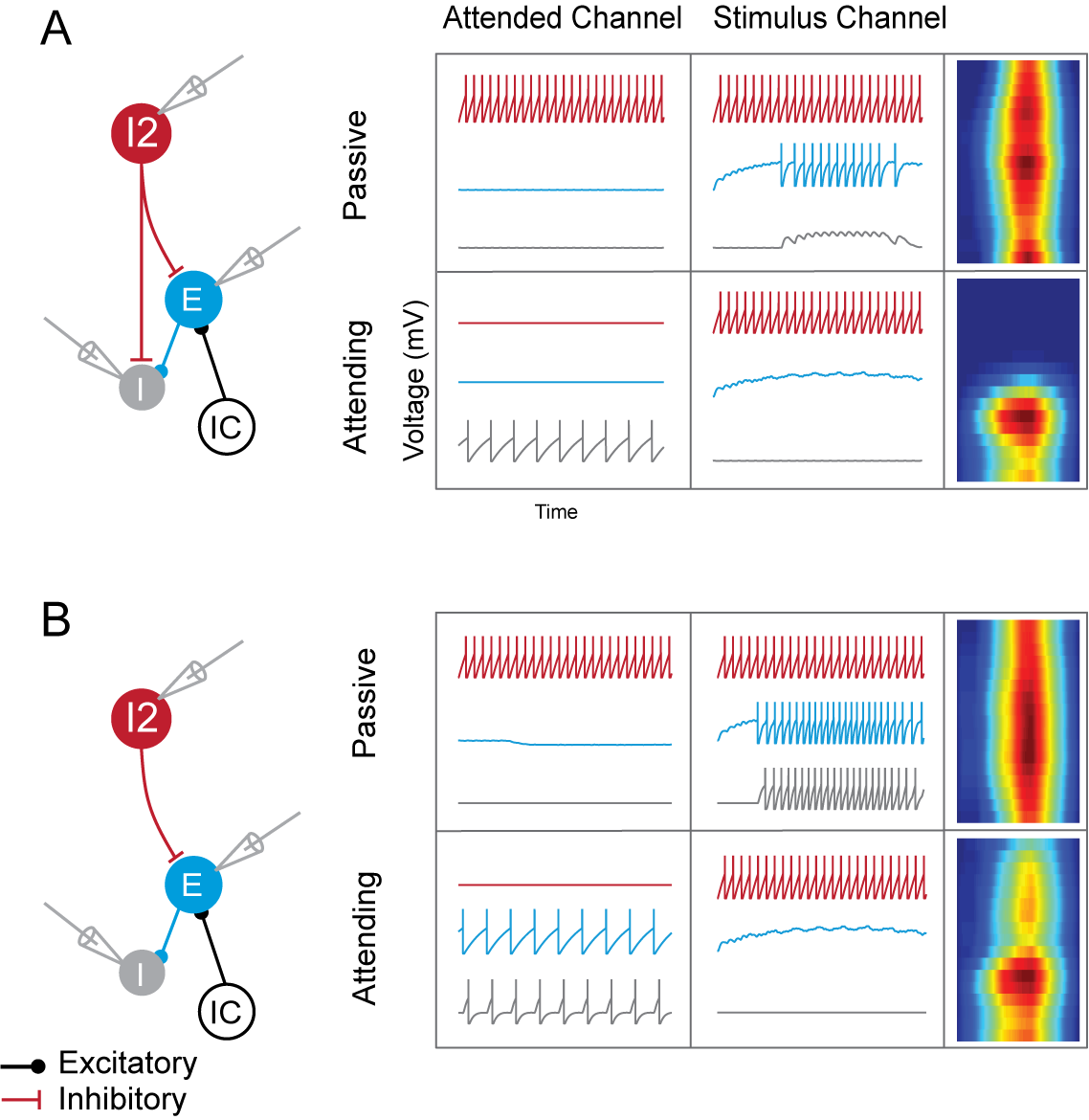

Supplement: S1 Fig — A) Direct inhibition from I2 to I neuron, and B) Feed forward inhibition from I2 -> E -> I neuron. Voltage traces of each model neuron under the passive or attending condition is shown on first two columns of the grid. The final column shows spatial tuning of the attended channel in the passive vs attending conditions. (TIF) [file pcbi.1009356.s001.tif]

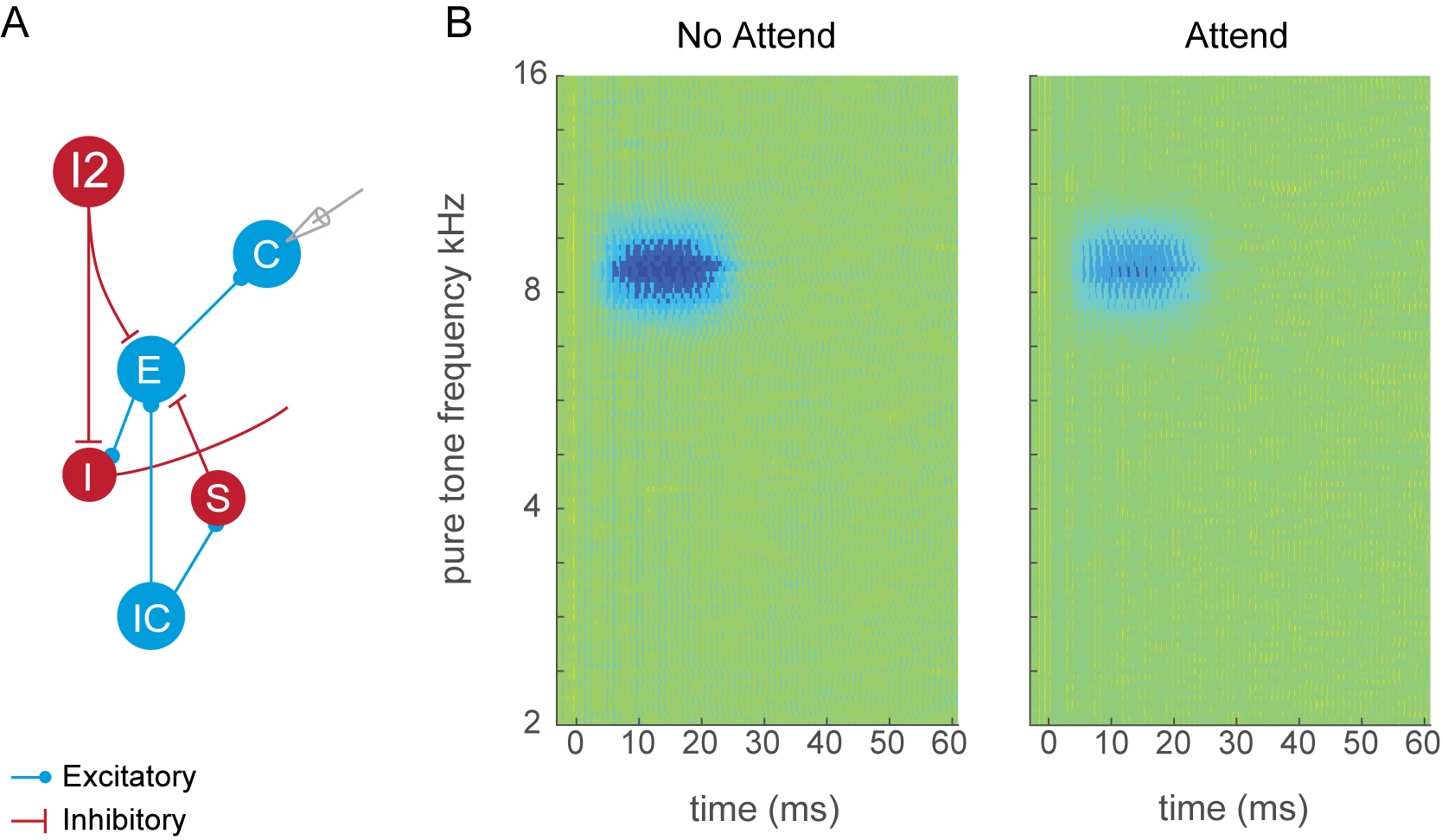

Supplement: S2 Fig — A) Within-channel inhibition (S neuron) is added to the AIM network for this simulation to induce suppression of activity relative to spontaneous firing of E neurons, thereby creating an inhibitory region in the frequency-dependent PSTH. Note that S neurons are distinct from I neurons, which inhibit other frequency channels. B) Frequency-dependent PSTHs. Blue regions in PSTH indicate lower firing rate relative to the spontaneous firing rate. When attention is turned on and I2 neuron is turned off, the E neuron is released from inhibition, resulting in the weakening of the inhibitory region. Simulation parameters are listed in the table below. (TIF) [file pcbi.1009356.s002.tif]
