# Supplementary material for: AIM: A network model of attention in auditory cortex
Source: PLoS Comput Biol. 2021 Aug 27;17(8):e1009356. doi: 10.1371/journal.pcbi.1009356 (PMC8462696; doi:10.1371/journal.pcbi.1009356)
Supplement: Attachment — Submitted filename: ResponsetoReviews.docx [file pcbi.1009356.s004.docx]

**Reply to Reviews.**

**We thank the reviewers for their comments and suggestions. We have addressed the issues raised by the reviewer. Below we reply point-by-point to the review.**

Reviewer #1: This paper presents a model for spectral and spatial tuning in the auditory cortex that is able to adjust this tuning based on a proposed attention mechanism. The model is inspired by known circuitry in cortex and may well be generalizable to other senses and regions of the brain. The spatial and spectral tuning essentially operate in the same way, with both represented as angle of incidence and tonotopic organizations across neurons, respectively. Temporal aspects such as modulation are not considered.

A major concern with the paper relates to attentional changes in spectral tuning. This is exemplified in Figure 4 where it is clear that the AIM model does not replicate the inhibitory regions of the STRFs seen in experimental studies. While it is claimed that the AIM model reproduces the experimental results, it is only reproducing half of the results, which significantly simplifies the problem. This hides the fact that, while the model produces an increase in response as well as a sharpening of the response for an excitatory region in the STRF, the model would also increase the response for an inhibitory region. Fritz and colleagues have shown in many publications that an excitatory region should be increased with attention, but an inhibitory region should be decreased, suppressed, or removed. Furthermore, David et al. (2012, [www.pnas.org/cgi/doi/10.1073/pnas.1117717109](http://www.pnas.org/cgi/doi/10.1073/pnas.1117717109)) showed that different training paradigms can produce the opposite effect, whereby an inhibitory region is increased, but an excitatory region is decreased, suppressed, or removed. The point here is that top-down control will have opposing effects on excitatory and inhibitory regions, but the mechanism proposed in this paper will increase both types of regions. It is a concern that this has not been mentioned or discussed.

**We now mention and discuss this issue in the discussion as suggested by the reviewer. We have also added a supplementary figure to illustrate that an inhibitory region can be decreased during attention, as shown by Fritz et al. We agree that modeling the effects of attention on inhibitory regions merit further investigation in the future. For example, the diverse effects of training paradigms on inhibitory regions will require modeling the effect of training, reward or punishment on cortical circuits in the model, which are outside the scope of this study.**

Introduction

The introduction is very sparse and does not provide much background information on the state of the art of modelling of the auditory system. While some other models are mentioned in the Discussion, there is value in highlighting the key achievements and shortcomings of existing models to inspire the development of the AIM approach.

**We have added a paragraph in the Introduction to provide more background information on modelling of the auditory system and to motivate the development of the AIM network.**

Results

The figures are generally rather difficult to interpret especially in comparing the model results with experimental results. The tuning plots for the AIM model and experimental results should be shown with the same time scales so that they can be more directly and easily compared. A key difference in spatial tuning (Fig 2) is that the duration of response to stimuli are very different between AIM and experiment. While I don’t think this is necessarily a problem, it is important to be able to easily see this and the differences explained.

**We have now changed the plots to have the same time scales so that they can be directly compared.**

Furthermore, it would be really useful to see peristimulus time histograms of the experimental data for direct comparison with the AIM output. Similarly, in Figs 4 and 5, the same suggestions apply.

**These plots were not part of the paper by Lee and Middlebrooks so, unfortunately, we could not include them. We have presented the model information in a similar format to that paper for ease of comparison.**

Line 131: Reference to Fig 4 should be Fig 3.

**Corrected.**

Figure 4: Explain what f_T and f_B are to help the reader.

**We have now added this explanation in the caption of Figure 4.**

Since the model is seeking to model many spatial channels, the functional implications of spatial attention would benefit with presentation of model results for less extreme differences than 90 degrees. While 0 and 90 could be used as an example, the quantitative results can be presented for many more angles to illustrate the strengths and limitation of the model in this regard.

**We have added these results in Fig. 6E and described them in the text (lines 248 – 252).**

The result for spectral attention is limited to the spectrum of the fundamental voicing frequency, F0, with a throwaway line that this may contribute to speech segregation. Further justification of this as a sufficient, or at least major, mechanism should be discussed especially in relation to the observations experimentally that the attentional mechanisms apply across a much wider range of frequency and that F0 is also largely a temporal phenomenon (seen as striations in spectrograms).

**We have deleted the line in the Results section. We give a more balanced statement in the discussion regarding the spectral aspect of F0 emphasizing that this is one of multiple components that may contribute to speech segregation. We now point out how the principle in the AIM network, illustrated for F0, could be applied to other features across a wider range of frequency, e.g., harmonics (Lines 449-456).**

**We believe a thorough analysis of temporal aspects of F0, will require incorporating temporal dynamics of responses in auditory cortex, e.g., synaptic depression and adaptation, which was outside the scope of this study. We now comment on this and suggest this as a future direction of investigation (Lines 457-465).**

The impact on the remaining spectral information should also be quantified in terms of how much the speech at higher frequencies well above F0 is being suppressed by the model.

**We have made a correction when calculating the baseline condition for the results shown in Figure 7. In the original figure, background yellow shaded regions were representative of the total subcortical response. We corrected this to represent the activity of the passive condition at the output of the AIM network. With this correction, the total spiking activities for the “passive” and “attend” conditions shown in the right-hand margins are placed on the same scale. Thus, the two curves can be compared directly to assess the increase or decrease in spiking activity as a function of frequency when compared to the baseline “passive” condition. As explained below, we have moderated our statements and elaborated the discussion to point out that this effect is one of several components that may influence speech segregation. With this clarification, we believe that a qualitative demonstration of the effect is sufficient.**

Furthermore, similar to the comment above, quantitative reporting across many speech tokens with different F0 could be reported including speech where F0 changes substantially across the duration of the token.

**Our goal in the manuscript was to illustrate how a unifying principle in the AIM network could be applied in the frequency domain in addition to the spatial domain. Based on the reviewers’ comments, we now realize that our presentation of these results in the context of speech segregation was too simplistic. We have moderated our discussion to point out that there are several other factors that could play an equal or more important role in speech segregation. Rather than present additional quantitative results on spectral F0 changes, which is likely to be an incomplete explanation, we instead discuss how the principle illustrated for F0 can be extended to other complex features, e.g., harmonics. We also suggest that future studies incorporate more complex features and temporal dynamics to investigate the implications for speech segregation.**

Discussion

The impact of not modelling the inhibitory regions of the STRFs should be discussed.

**We have now added a paragraph to address this in the discussion (Line 441-448).**

On p. 17, a case is made that there is a distinction between the type of inhibition used in AIM vs. “classical” lateral inhibition. I suggest that discussion should be added of WHY this is important to consider.

**We have now added this to the discussion (Line 380-390).**

Methods

The neural model is poorly described. There are many variables that are not included in the formulation that is presented, including i_tonic, V_thresh, V_spike, V_reset, i_e, as well as the formulation of netcon. What is the spiking-and-reset mechanism and how it is handled? Why is there an action potential voltage for a simply LIF model? The synaptic input current equation includes u(t), which is zero for t<0 and positive otherwise and so does not represent events occurring at t > 0. I can understand how it is used but the notation is imprecise. I suggest that labels use non-italics, reserving italics only for variables and functions. Table 1 include 1/10 for g_leak – it is not clear why it is given in this way.

**We thank the reviewer for pointing out these issues. We removed V_spike since it was not necessary in the final integra-and-fire model and we removed the unused I_tonic term. We added the description of spike-and-reset mechanism and expanded the description of the network connectivity (netcon) formulation. We clarified that t represents the time since the occurrence of previous spike. The u(t) term ensures that EPSPs have non-zero values after the previous spike has occurred. We removed italics from column labels.**

The functional listening method does not describe how the measurements of outcomes were made.

**We have now revised the Methods section to incorporate the reviewer’s suggestions and address the reviewer’s comments (601-605).**

Reviewer #2: The authors propose a network model for auditory attention, with a mechanism called attentional inhibitory modulation (AIM), in which an additional group of inhibitory neurons (called I2) are added to the conventional E-I networks and could emulate spatial and spectral auditory attention through disinhibition of the E-I network. In particular, they consider spectral selectivity, spectral sharpening, secondary spectral selectivity, and a cocktail party scenario, and replicate some of the existing experimental findings using the AIM mechanism. They further hypothesize that the top-down inputs could come from VIP neurons, I2 neurons could be SOM neurons, and I neurons could be SOM or PV neurons. Also, the different "modes" of the network are achieved by neuromodulatory mechanisms such as those mediated by acetylcholine and norepinephrine.

This is a nice theoretical contribution and the predictions of the E-I network enhanced with the AIM mechanism are impressive in replicating various existing phenomenological findings involving auditory attention. I have the following comments/suggestions for the authors:

1) The hypothesis that switching from the "passive"/"monitor" to "attentive" mode is facilitated by modulatory mechanisms (e.g., cholinergic) is relevant to long-term effects, i.e., the time constants of ACh and NE mechanisms are in the order of minutes. However, switching from passive to attentive could happen in the order of seconds or sub-second. Could the author comment on potential mechanisms that could result in rapid changes of in the top-down input (i.e., VIP neurons) as well? Could it be the case that the top-down input is coming from SOM neurons, and the VIP neurons control the top-down input?

**We now comment on potential mechanisms that could result in rapid changes in the top-down input to VIP neurons in the Discussion (Line 338-348). Specifically, one potential mechanism is via top down input from frontal areas to auditory cortex, which form a cortico-cortical loop. Such inputs can affect auditory cortical processing on the time-scale of hundreds of milliseconds.**

**Although possible, we could not formulate a specific scenario where the top-down input arises from SOM neurons in a manner that is consistent with the model and existing literature.**

2) Somehow related to the previous comment, given that the underlying neurons are modeled as LIF, it would be helpful to see how "turning off" an I2 neuron at a specific angle (in the spatial model) or frequency would transiently change the tuning properties. I suggest adding some snapshots of the STRFs vs. time after a particular I2 neuron is turned off, corresponding to the results of Figs. 2, 4, and 5.

**This is an interesting idea and a great suggestion. However, we feel that modeling such dynamics would require including more realistic models of cortico-cortical loops/top-down inputs from frontal areas as well as temporal dynamics, including synaptic depression and adaptation, which are outside the scope of the current study. We suggest future improvements to the AIM model include these temporal mechanisms (Line 457-465).**

3) How are the STRFs in Figs. 2, 4, and 5 estimated? Are they estimated based on multiple repeated trials and using reverse correlation on the C responses? Please explain.

**Our goal was to construct a unified model for both the spatial and frequency domains and compare to experimental data from both cases. Thus, we presented the responses of the cortical C neuron as azimuth or frequency-dependent peristimulus time histograms (PSTHs). These model PSTHs were then compared to the analogous azimuth dependent PSTHs in the study by Lee and Middlebrooks; and, as an approximation, to the STRFs in the studies by Fritz et al and Atiani et al. We now clarify this in the results and explain their calculation in the Methods.**

4) In the cocktail party simulation (Figs. 6 and 7), the model of attending to the male vs. female F0 seems to be too simplistic, given that a growing list of studies suggest that tracking the slow spectrotemporal modulations of speech (e.g., envelope) are key in maintaining auditory attention. Could the authors use a similar model, but consider the average of spectral power across channels as the input to neurons, instead of just F0?

**Our goal in the manuscript was to illustrate how a unifying principle in the AIM network could be applied in the frequency domain in addition to the spatial domain. Based on the reviewers’ comments, we now realize that our presentation of these results in the context of speech segregation was too simplistic. We have moderated our discussion to point out that there are several other factors that could play an equal or more important role in speech segregation.**

**We believe a thorough analysis of temporal aspects, such as tracking the slow spectrotemporal modulations of speech, will need to incorporate temporal dynamics of responses in auditory cortex, e.g., synaptic depression and adaptation, which was outside the scope of this study. We now comment on this and suggest this as a future direction of investigation.**

5) Also in the cocktail party setting, how are the I to E connection profiles chosen? Is the I to E connectivity determined based on an assumed tonotopic map/preference, or just random connections? Please clarify.

**We now clarify this in the methods section (Line 593-595).**

6) In lines 490-492, it is stated that the effect of ACh receptors is modeled by changing the global gain of E-C and IC-E connections. How are these gains (i.e., 0 and 2.5) determined? If these changes are not applied, or other gain values are used, would the resulting spectral tuning profiles change dramatically? It seems that the top-down signal to I2 neurons suffices for the network model to exhibit the changes in tuning. Could the authors explain why these global gain changes are necessary here?

**We now explain how the gain values are determined (Line 570-574). The global gain changes were used to model experimentally observed cholinergic mechanisms. Specifically, we modeled both muscarinic changes in intracortical connections and nicotinic changes in thalamocortical connections. We compare this mechanism to top-down selective attention mechanism in the AIM network and discuss these alternative/additional mechanisms (Lines 325-337).**
