# Supplementary material for: AIM: A network model of attention in auditory cortex
Source: PLoS Comput Biol. 2021 Aug 27;17(8):e1009356. doi: 10.1371/journal.pcbi.1009356 (PMC8462696; doi:10.1371/journal.pcbi.1009356)
Supplement: Attachment — Submitted filename: ResponsetoReviews2_KC_KS_final_clean.docx [file pcbi.1009356.s005.docx]

**Reply to Reviews.**

**We thank the reviewers for their comments and suggestions on the revised manuscript. Reviewer 2 noted that we addressed all of the reviewer’s previous comments. We have now addressed the remaining issues raised by Reviewer 1. Based on reviewer comments, we have made minor points of clarification throughout the manuscript. Below we reply point-by-point to the review.**

Reviewer #1: Most of my concerns have been addressed well in this revised version of the manuscript.
My major remaining concern is that the model is not able to replicate the complex STRFs as seen in experiments, which include both excitatory and inhibitory sub-regions. The additional supplementary figure shows how an inhibitory-only STRF can be modified by the top-down influences, which is good, but actually further highlights this shortcoming of the model. This is despite the Author Summary stating that the model can account for "diverse" experimental observations, which implies a much more complete model. It is not clear how the model might be expanded to be able to model experimental data with the greater level of detail.

**We have removed the word “diverse” from the author summary. In the manuscript, we demonstrated how our model explains attentional changes in excitatory regions of STRFs, and in the supplementary material, we demonstrated how our model can also explain attentional changes in an inhibitory region of a STRF. We hypothesize that complex STRFs can be obtained by combining the techniques used in these demonstrations. Our main focus, however, is on attentional mechanisms in the cortex, and this can be more clearly illustrated with simple excitatory and inhibitory regions, without the use of complex STRFs. We agree that our simulations are a simplification** **and that complex STRFs should be modeled in future investigations. We have now suggested this in the discussion (Line 450-451).**

The plots in Figure 2 are better with the same time scales. However, this further highlights the differences in temporal durations of the responses between the model and the Lee and Middlebrooks data. I asked for these duration differences to be explained but this was not addressed in the response or revised manuscript.

**We now clarify in the results that we are specifically modelling the sharpening of spatial tuning (lines 126-127) using integrate-and-fire neurons (line 108) . We also note and explain the differences in response durations between the model and the study by Lee and Middlebrooks in the discussion (Lines 462-468).**

I like the addition of Fig. 6E. However, it is very hard to understand this figure without very careful re-reading and I still am not sure that I understand it. I advise a fuller explanation be added of the method for creating the figure and text to help the reader interpret the figure.

**We thank the reviewer for pointing this out. We have clarified the description of Fig 6E in the results (Lines 250-255), figure caption (Lines 269-271), and methods (Lines 605-617) sections.**

There are a number of typos in the paper that need to be fixed, e.g.:
line 33: in [the] midst
70: an animal [is]
76: compute salience
many semicolons are used where commas would be more appropriate

**We have fixed these typos and replaced many semicolons with commas throughout the manuscript.**

Reviewer #2: The authors have done a great job addressing my previous comments/suggestions. I have no further comments at this point.
